# Connexin43 peptide, TAT-Cx43_266–283_, selectively targets glioma cells, impairs malignant growth, and enhances survival in mouse models in vivo

**DOI:** 10.1093/neuonc/noz243

**Published:** 2019-12-28

**Authors:** Myriam Jaraíz-Rodríguez, Rocío Talaverón, Laura García-Vicente, Sara G Pelaz, Marta Domínguez-Prieto, Andrea Álvarez-Vázquez, Raquel Flores-Hernández, Wun Chey Sin, John Bechberger, José M Medina, Christian C Naus, Arantxa Tabernero

**Affiliations:** 1 Department of Biochemistry and Molecular Biology, Institute of Neurosciences Castilla y León (INCYL), University of Salamanca, Salamanca, Spain; 2 Department of Cellular and Physiological Sciences, The Life Sciences Institute, University of British Columbia, Vancouver, British Columbia, Canada

**Keywords:** cell-penetrating peptides, connexin, glioma, Src

## Abstract

**Background:**

Malignant gliomas are the most frequent primary brain tumors and remain among the most incurable cancers. Although the role of the gap junction protein, connexin43 (Cx43), has been deeply investigated in malignant gliomas, no compounds have been reported with the ability to recapitulate the tumor suppressor properties of this protein in in vivo glioma models.

**Methods:**

TAT-Cx43_266–283_ a cell-penetrating peptide which mimics the effect of Cx43 on c-Src inhibition, was studied in orthotopic immunocompetent and immunosuppressed models of glioma. The effects of this peptide in brain cells were also analyzed.

**Results:**

While glioma stem cell malignant features were strongly affected by TAT-Cx43_266–283_, these properties were not significantly modified in neurons and astrocytes. Intraperitoneally administered TAT-Cx43_266–283_ decreased the invasion of intracranial tumors generated by GL261 mouse glioma cells in immunocompetent mice. When human glioma stem cells were intracranially injected with TAT-Cx43_266–283_ into immunodeficient mice, there was reduced expression of the stemness markers nestin and Sox2 in human glioma cells at 7 days post-implantation. Consistent with the role of Sox2 as a transcription factor required for tumorigenicity, TAT-Cx43_266–283_ reduced the number and stemness of human glioma cells at 30 days post-implantation. Furthermore, TAT-Cx43_266–283_ enhanced the survival of immunocompetent mice bearing gliomas derived from murine glioma stem cells.

**Conclusion:**

TAT-Cx43_266–283_ reduces the growth, invasion, and progression of malignant gliomas and enhances the survival of glioma-bearing mice without exerting toxicity in endogenous brain cells, which suggests that this peptide could be considered as a new clinical therapy for high-grade gliomas.

**Key Points**

1. Connexin43 peptide, TAT-Cx43_266–283_, selectively targets glioma cells.
2. TAT-Cx43_266–283_ impairs malignant glioma growth and enhances survival in mouse models in vivo.

**Importance of the Study**

Malignant gliomas are the most frequent and aggressive primary brain tumors. Here, we reveal that TAT-Cx43_266–283_, an inhibitor of Src based on connexin43, selectively targets glioma cells, impairs the growth of these tumors in vivo, and enhances the survival of glioma-bearing mice. While glioma cells were strongly affected by TAT-Cx43_266–283_, the toxicity in neurons and astrocytes was notably lower than that found with dasatinib, another c-Src inhibitor currently used in glioma clinical trials. Intraperitoneally administered TAT-Cx43_266–283_ decreased the invasion of intracranial tumors generated by murine glioma cells in immunocompetent mice. When human glioma stem cells were intracranially injected with TAT-Cx43_266–283_ into immunosuppressed mice, there was reduced expression of stemness markers and reduced tumorigenicity of these glioma cells. Furthermore, TAT-Cx43_266–283_ enhanced the survival of immunocompetent mice bearing gliomas derived from murine glioma stem cells. Our results highlight the importance of this compound for the design of new therapies against gliomas.

Malignant gliomas are among the most incurable cancers, with a median survival rate of 1–2 years.^1^ These tumors are composed of a heterogeneous population of cells, including many with stem cell–like properties, called glioma stem cells (GSCs). GSCs are characterized by their self-renewal capacity, high oncogenic potential, resistance to standard therapies,^2,3^ and high invasive capacity.^4,5^

Connexin43 (Cx43) is a protein that forms gap junction channels and hemichannels playing important roles in cellular communication.^6^ Cx43 interacts with a plethora of intracellular signaling partners,^7^ such as c-Src,^8^ which is a proto-oncogene that regulates a wide range of cellular events related to cancer.^9–11^ Indeed, in astrocytes, ectopic expression of v-Src, the active viral form of Src, promotes the development of astrocytomas.^12^

In the context of gliomas, Cx43 has been traditionally considered a tumor suppressor protein because it is downregulated in malignant glioma cells,^13–16^ including GSCs,^17–19^ compared with astrocytes,^20^ and because the ectopic expression of Cx43 in glioma cells reduces their rate of proliferation^21^ and tumor formation in vivo.^22^ Among other mechanisms, Cx43 exerts its antitumor effects through the recruitment of c-Src together with its endogenous inhibitors, C-terminal Src kinase (CSK) and phosphatase and tensin homolog (PTEN), which inhibit the activity of c-Src.^23,24^ Because c-Src is overactivated in malignant gliomas,^25^ the ectopic expression of Cx43 might have beneficial effects on glioma therapy. However, connexins can also play pro-tumorigenic roles in glioma.^25^ Indeed, Cx46 is upregulated in GSCs and required for their maintenance.^17^ In addition, restoration of Cx43 in glioma cells can favor glioma invasion.^26,27^ These studies indicate that very specific Cx-related tools should be used to target glioma.

Consequently, instead of the whole protein we used the region of Cx43 responsible for Src inhibition^18^ (amino acids 266 to 283), fused to the transactivator of transcription (TAT) cell-penetrating sequence (TAT-Cx43_266–283_). We found that TAT-Cx43_266–283_ retained the ability to recruit c-Src, CSK, and PTEN,^24^ leading to c-Src inhibition in a broad spectrum of glioma cells. TAT-Cx43_266–283_, by inhibiting c-Src, reduces the expression of markers of stemness, such as Sox2, neurosphere formation, proliferation, migration, and invasion in GSCs, including primary GSCs derived from patients and freshly removed surgical specimens.^18,28^

However, TAT-Cx43_266–283_ toxicity in nontumor cells and in vivo effects remain unknown, hampering potential clinical applications. Therefore, in this study we explored the effects of TAT-Cx43_266–283_ in neurons and astrocytes and compared them with those of other c-Src inhibitors currently being evaluated in clinical trials (https://clinicaltrials.gov/ct2/show/NCT00895960, https://clinicaltrials.gov/ct2/show/NCT00423735, and https://clinicaltrials.gov/ct2/show/study/NCT00892177). Once we confirmed the lack of toxic effects of TAT-Cx43_266–283_ in healthy brain cells, we investigated the antitumor effects of TAT-Cx43_266–283_ in vivo in 3 mouse models of glioma using mouse glioma cells, mouse GSCs, and human GSCs.

## Methods

For details of media and protocols, see the Supplementary Material.

### Animals

Albino Wistar rats, nonobese diabetic/severe combined immunodeficient (NOD/SCID) mice, and C57BL/6 mice were obtained from the animal facility of the University of Salamanca and the University of British Columbia. The animal procedures were approved by the ethics committee of the University of Salamanca and the Junta de Castilla y León and were carried out in accordance with European Community Council directives (2010/63/UE), Spanish law (RD 53/2013 BOE 34/11370–420, 2013) for the use and care of laboratory animals, and the University of British Columbia Animal Care Committee (protocol number: A14-0183) and performed in accordance with the guidelines established by the Canadian Council on Animal Care.

### Cell Culture

Primary astrocytes and neurons were cultured as described.^29^ Human G166 GSCs were obtained from Dr Steven Pollard (Medical Research Council Centre for Regenerative Medicine, University of Edinburgh) or BioRep (Milan, Italy) and cultured in adherent conditions.^28^ For the indicated experiments, cells were labeled with 2 µL/mL PKH26 (Red Fluorescent Cell kit, Sigma-Aldrich) for 5 min. GL261-GSCs were obtained from GL261 cells as previously described.^30^

For GSC-astrocyte cocultures, 25 000 GSCs/cm^2^ were cultured on top of confluent astrocytes and allowed to integrate for 72 h.

Mouse GL261 glioma cells (National Cancer Institute–Frederick Division of Cancer Treatment and Diagnosis) and GSCs were transfected with pcDNA-mCherry plasmid using Lipofectamine 2000 (Invitrogen) as described.^26^

### Organotypic Brain Slice Cultures

Organotypic brain slice cultures were prepared as described.^31^ 350-μm-thick newborn rat brain slices were cultured onto cell culture inserts for 19–20 days in vitro. For GSC-organotypic brain slice cocultures, 2500 PKH26-labeled GSCs were placed onto each brain slice and allowed to engraft for 2 days prior to the experiment.

### Intracranial Implantation of Glioma Cells

One microliter of saline containing 5000 cells was injected by a unilateral stereotaxic intracerebral injection into the right cortex with a Hamilton microsyringe. Murine mCherry-GL261 glioma cells or murine mCherry-GL261-GSCs were injected into C57BL/6 while human GSCs labeled with PKH26 were injected into NOD/SCID mice. At the indicated times, animals were transcardially perfused and brains were processed to obtain 20- to 40-µm-thick coronal sections.

### Treatments

Peptides (>85% pure) were obtained from GenScript. For ex vivo and in vitro studies, the peptides and the c-Src inhibitor dasatinib (Selleck Chemicals) were used at the specified concentrations in culture medium at 37°C for the indicated times.

For in vivo studies, saline, TAT, or TAT-Cx43_266–283_ were intraperitoneally injected or intracranially co-injected with GSCs at the specified concentrations and times.

### Immunofluorescence

For in vitro and ex vivo studies, samples were subjected to immunocytochemistry for glial fibrillary acidic protein (GFAP), microtubule-associated protein 2 (MAP-2), human nestin, and 4′,6′-diamidino-2-phenylindole (DAPI) or TO-PRO-3 as previously described.^18^ For in vivo studies, tumor sections were subjected to immunohistochemistry for human nestin, Sox2, Stem121, PTEN, Src (Y416), and activated caspase-3.

### MTT Assay

Cells were incubated with culture medium containing 0.5 mg/mL MTT (3-(4,5-dimethylthiazol-2-yl)-2,5-diphenyltetrazolium bromide) (Sigma-Aldrich). After 10 min in dimethyl sulfoxide (DMSO), the absorbance was measured at 570 nm.

### Matrigel Invasion Assay

750 000 cells were plated into the upper chamber and were allowed to invade Matrigel for 15 h. Cells in the lower surface were counted in 10 random fields per insert.

### Quantification of the Invasiveness of Intracranial Tumors

The invasiveness of mCherry-GL261 cells was determined using Fiji software by quantifying the fractal dimension (D) of the tumor rims.^32,33^ We blindly determined the D by thresholding the mCherry fluorescence images, establishing the tumor rims, and quantifying D using FracLac (Fiji) in at least 3 separate sections per animal.

### Statistical Analyses

Student’s *t*-test was used when 2 groups were compared. For more than 2 groups, one-way ANOVA was used, followed by a post hoc test (Tukey). The survival of glioma-bearing mice was analyzed by Kaplan–Meier curves and differences were compared by log-rank test analysis.

## Results

### TAT-Cx43_266–283_ Selectively Targets GSCs Without Toxic Effects in Neurons and Astrocytes

**Fig. 1:**
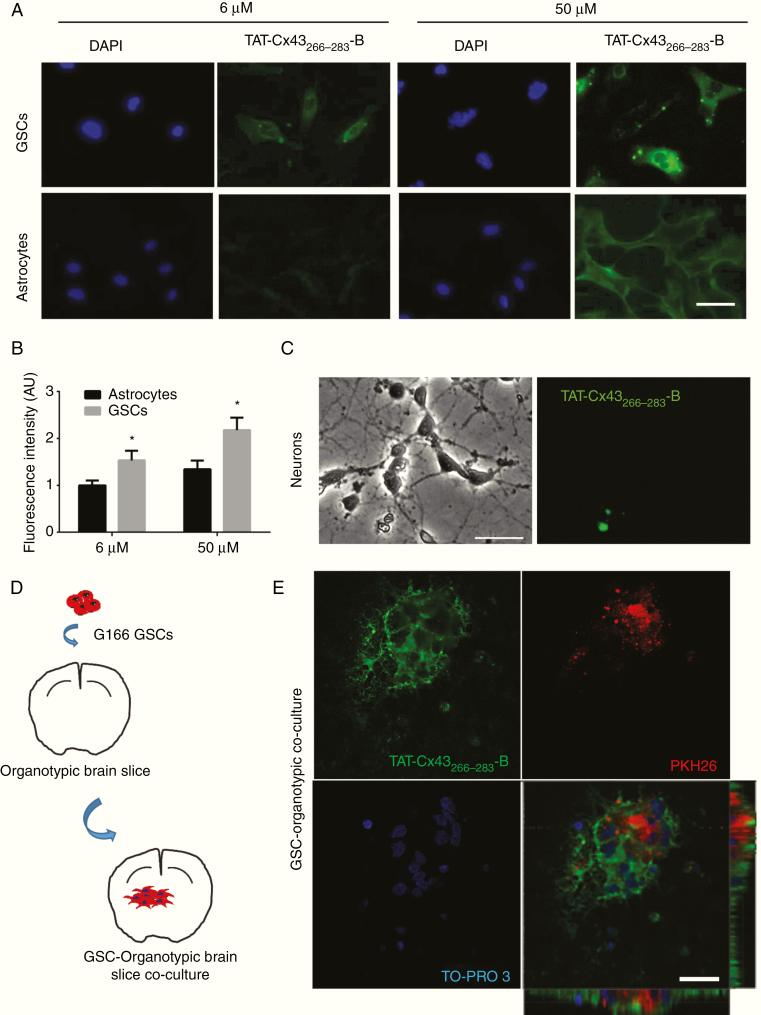
TAT-Cx43_266–283_ internalization in GSCs, neurons, and astrocytes. (A–C) Cells incubated with TAT-Cx43_266–283_ fused to biotin at the C-terminus (TAT-Cx43_266–283_-B) for 5 min and revealed with streptavidin. TAT-Cx43_266–283_ (green) and DAPI (blue) staining of the same field. (A) Internalization of 6 and 50 µM TAT-Cx43_266–283_-B in GSCs and astrocytes. Bar: 100 µm. (B) Quantification of TAT-Cx43_266–283_-B fluorescence expressed as arbitrary units (AU) and the mean ± SEM of 3 independent experiments (*t*-test: *P < 0.05). (C) Images from the same field showing the lack of internalization in neurons incubated with 50 µM of TAT-Cx43_266–283_-B for 5 min. Bar: 50 µm. (D) Scheme for GSC–organotypic brain slice cocultures. (E) Cocultures incubated with 50 µM TAT-Cx43_266–283_-B for 5 min. TAT-Cx43_266–283_-B internalization (green) in GSCs (PKH26 [red]) and surrounding cells (nuclear staining, TO-PRO-3 [blue]). Bar: 25 µm. See also Supplementary Movie 1 showing the complete Z-stack.

To analyze TAT-Cx43_266–283_ internalization, GSCs, astrocytes, and neurons in culture were exposed to increasing concentrations of TAT-Cx43_266–283_ fused to biotin (TAT-Cx43_266–283_-B) for 5 min. TAT-Cx43_266–283_-B was internalized by astrocytes and more efficiently by GSCs, but not significantly by neurons (Fig. 1A–C and Supplementary Figure 1). To confirm these results, we used an ex vivo model of brain tumors.^34,35^ PKH26-labeled GSCs were placed into brain slices (Fig. 1D), which were incubated with TAT-Cx43_266–283_-B for 5 min. TAT-Cx43_266–283_-B was internalized by GSCs within the brain parenchyma and by brain cells surrounding tumor cells (Fig. 1E; for complete Z-stack, see Supplementary Movie 1).

**Fig. 2:**
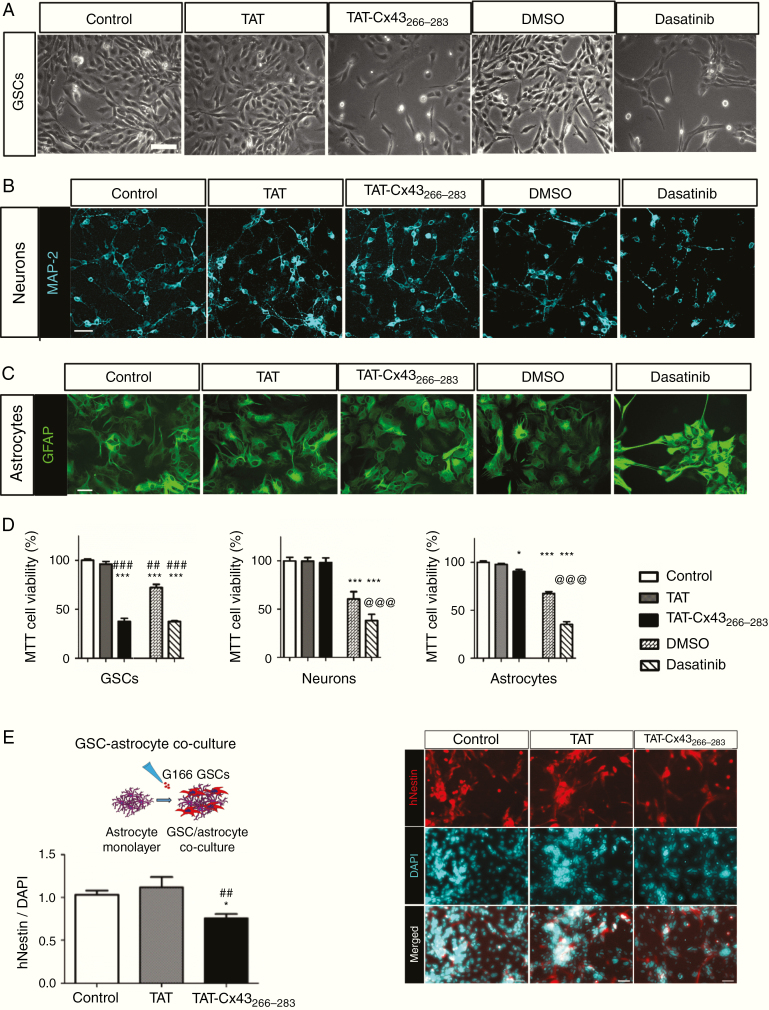
Effect of TAT-Cx43_266–283_ and dasatinib on cell viability in GSCs, neurons, and astrocytes. Cells treated with 50 µM TAT, 50 µM TAT-Cx43_264-283_, 1 µM of the c-Src inhibitor dasatinib, or 0.1% (v/v) DMSO (vehicle for dasatinib) for 72 h. Three independent experiments were carried out with similar results. (A) Images showing the reduction in GSC viability induced by TAT-Cx43_264-283_ and dasatinib. Bar: 100 µm. (B) Immunofluorescence for MAP-2 (turquoise) in neurons showing a reduction in MAP-2 by dasatinib. Bar: 50 µm. (C) Immunofluorescence for GFAP (green) in astrocytes showing an alteration induced by dasatinib. Bar: 50 µm. (D) MTT results expressed as the percentage of the control; mean ± SEM; *n* ≥ 3 (ANOVA: *P < 0.05, ***P < 0.001 vs control; #*P* < 0.05, ##*P* < 0.01, ###*P* < 0.001 vs TAT; @@@*P* < 0.001 vs DMSO). (E) GSC–astrocyte cocultures treated with 50 µM TAT or TAT-Cx43_266–283_ for 72 h. Images showing human nestin (hNestin, red) expressed by GSCs and DAPI nuclear staining (turquoise) of GSCs and astrocytes (right). Bar: 50 µm. Left, quantification of the nestin-positive cells vs DAPI (total cells); mean ± SEM; *n* = 3 (ANOVA: *P < 0.05 vs control and ##*P* < 0.01 vs TAT).

In agreement with our previous results,^24,28^ TAT-Cx43_266–283_ reduced GSC growth (Fig. 2A). However, we observed no differences in the morphology of neurons (Fig. 2B and Supplementary Figure 2A) or astrocytes (Fig. 2C and Supplementary Figure 2B). In fact, the expression of MAP-2 and GFAP, neuronal and astrocytic markers, respectively, was not affected by TAT-Cx43_266–283_ (Fig. 2B, C). Moreover, time-lapse imaging of neurons in culture showed that neuron and neurite development was very similar among control, TAT-, or TAT-Cx43_266–283_–treated cultures (Supplementary Movies 2, 3, and 4, respectively). TAT-Cx43_266–283_ reduced GSC viability by about 60% (Fig. 2D), whereas neuronal viability remained unaffected and astrocyte viability was only slightly decreased (by about 15%). To confirm these results, we analyzed the effect of TAT-Cx43_266–283_ in a GSC-astrocyte coculture. Our results showed that TAT-Cx43_266–283_ specifically decreased the growth of GSCs (Fig. 2E), because the number of cells expressing human nestin over the total number of cells decreased by about 25%.

Because the effect of TAT-Cx43_266–283_ is exerted through inhibition of the oncogenic activity of c-Src,^18^ we compared the effect of TAT-Cx43_266–283_ with that of dasatinib, a well-known inhibitor of this tyrosine kinase.^36^ Both exerted a similar reduction in GSC growth (about 60%; Fig. 2A, D). However, while TAT-Cx43_266–283_ did not affect neurons or astrocytes, dasatinib affected neuronal and astrocyte morphology, reducing MAP-2–positive neurites (Fig. 2B) and increasing GFAP staining (Fig. 2C), suggesting neuronal damage and increased astrocytic reactivity, respectively. MTT assays confirmed that dasatinib reduced the viability of neurons and astrocytes by about 30% and 50%, respectively (Fig. 2D).

We have shown that TAT-Cx43_266–283_ strongly reduces the migration and invasion of GSCs through inhibition of c-Src and focal adhesion kinase (FAK).^28^ The present study revealed that TAT-Cx43_266–283_ did not modify neuron or astrocyte migration, as shown by neuronal motility (Supplementary Movies 2, 3, and 4) and by wound-healing assays performed in astrocytes (Supplementary Figure 3A, B). This is consistent with the lack of effect of TAT-Cx43_266–283_ on FAK activity found in astrocytes, in contrast to the effect in glioma cells^28^ (Supplementary Figure 3C). However, dasatinib significantly reduced astrocyte migration (Supplementary Figure 3A, B).

Altogether, these data suggest a specific effect of TAT-Cx43_266–283_ on GSCs, with lower toxicity in healthy brain cells than another c-Src inhibitor.

### TAT-Cx43_266–283_ Reduces the Invasion of GL261 Glioma Cells In Vivo

To address the effects of TAT-Cx43_266–283_ on glioma invasion in vivo, we selected the same model that showed a pro-invasive effect of Cx43^26^ consisting in the intracranial injection of mCherry-GL261 cells in C57BL/6 mice.

**Fig. 3:**
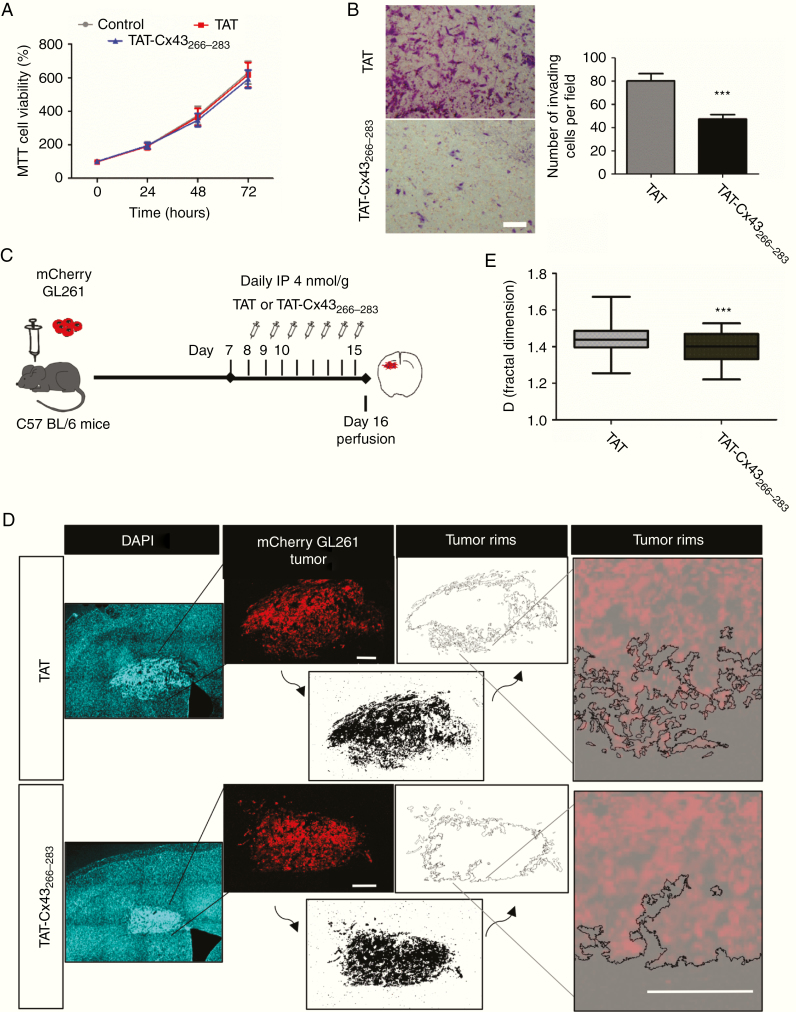
TAT-Cx43_266–283_ reduces the invasion of GL261 glioma cells in vivo. (A) MTT of GL261 cells incubated with 100 µM TAT or TAT-Cx43_266–283_. Percentage of the control (mean ± SEM; *n* = 3; ANOVA). (B) Matrigel invasion assay. GL261 cells were treated with 100 µM TAT or TAT-Cx43_266–283_ for 15 h. Representative images of the inserts and quantification. Bar: 100 µm; mean ± SEM; *n* = 3 (Student’s *t*-test: ***P < 0.001). (C) GL261 cells were intracranially implanted in syngeneic mice (C57BL/6) and allowed to grow for 7 days. Then, a daily i.p. injection of 4 nmol/g TAT or TAT-Cx43_266–283_ was administered for the next 7 days. (D) Mosaic immunofluorescence of DAPI (blue), mCherry GL261 glioma cells (red), the thresholded images (bottom, black), the tumor rims (top), and their magnifications. Bars: 200 µm. (E) The box plot shows the fractal dimension (D) of the tumor rims. D was determined in at least 3 separate sections per animal in 12 TAT and 13 TAT-Cx43_266–283_ animals from 4 independent experiments. The band inside the box represents the median and the bottom and top of the box represent the first and third quartiles. The top and bottom whiskers reflect the minimum and maximum values, respectively (Student’s *t*-test: ***P < 0.001).

First, we analyzed the effect in vitro. While mCherry-GL261 cell growth was not modified (Fig. 3A), cell invasion was strongly reduced by TAT-Cx43_266–283_ (Fig. 3B), which is consistent with the reduction in FAK activity (Supplementary Figure 3C)_._ To analyze the effect in vivo, 1 week after tumor implantation TAT-Cx43_266–283_ was intraperitoneally injected (Fig. 3C). After 15 days, TAT-Cx43_266–283_ reduced the complexity of the tumor borders (Fig. 3D). Indeed, TAT-Cx43_266–283_ significantly reduced the fractal dimension values of the tumor borders (Fig. 3E), an index of tumor invasion,^32^ suggesting a reduction in tumor invasion. Although no effects were found in PTEN expression in vitro (Supplementary Figure 3C) and in vivo (Supplementary Figure 4A, B), the activity of Src (Y416 Src) was reduced by TAT-Cx43_266–283_ in vivo (Supplementary Figure 4C), indicating that TAT-Cx43_266–283_ reduced the activity of the Src–FAK axis with the subsequent effects in glioma cell invasion in vivo.

### TAT-Cx43_266–283_ Reverses the Human GSC Phenotype and Reduces GSC Tumorigenicity In Vivo

**Fig. 4:**
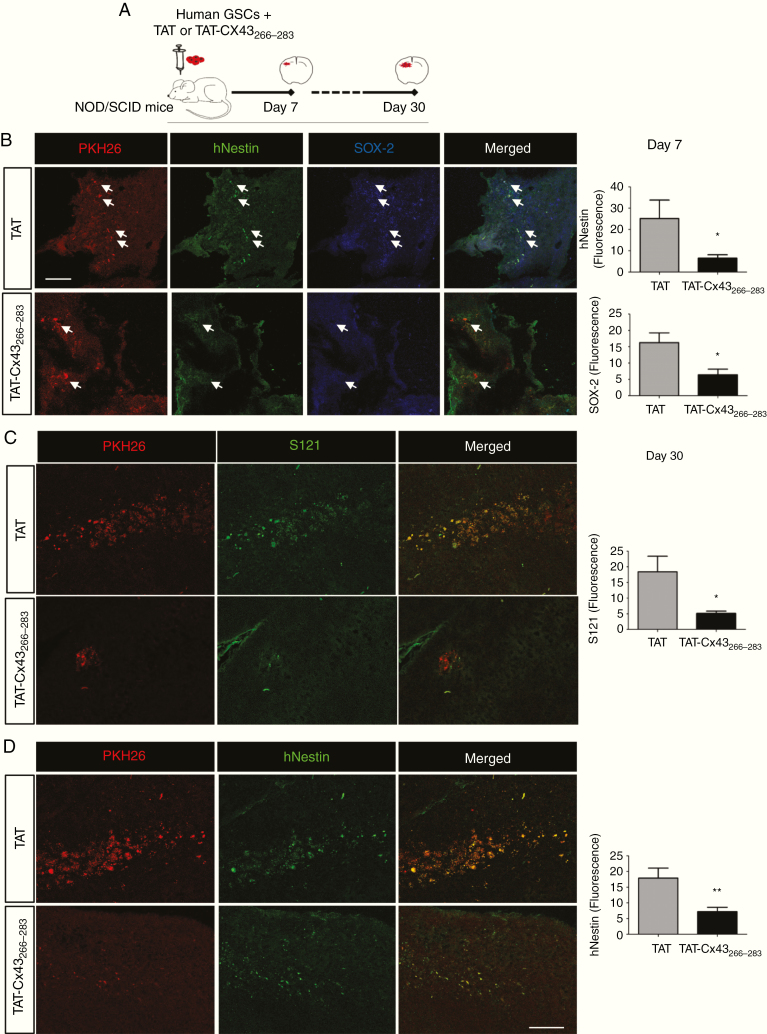
TAT-Cx43_266–283_ reduces Sox2 and human nestin expression and tumorigenicity in human GSCs intracranially implanted into mice. (A) Human PKH26-labeled GSCs were intracranially injected together with 100 µM TAT or TAT-Cx43_266–283_ in NOD/SCID mice. After 7 (B) or 30 (C and D) days, brain sections were analyzed. (B) PKH26 (red), human nestin (hNestin; green), Sox2 (blue), and merged images of the same field. White arrows indicate some PKH26-labeled cells to illustrate the reduction in hNestin and Sox2 expression in these cells after their treatment with TAT-Cx43_266–283_ for 7 days_._ Bar: 75 µm. Quantification of hNestin and Sox2 fluorescence intensity. (C and D) Images showing a reduction in PKH26, Stem121 (S121), and hNestin after treatment with TAT-Cx43_266–283_ for 30 days. Bar: 75 µm. Quantification of S121 and hNestin fluorescence (mean ± SEM). Between 2 and 5 sections per animal and 4 or 5 animals per condition from 3 independent experiments were analyzed (Student’s *t*-test: *P < 0.05, **P < 0.01).

To address the effect of TAT-Cx43_266–283_ on the stemness of human GSCs in vivo, PKH26-labeled human GSCs were intracranially injected together with TAT or TAT-Cx43_266–283_ into the brains of NOD/SCID mice (Fig. 4A). Seven days after implantation, the levels of Sox2 and human nestin were analyzed in GSCs (Fig. 4B). We found that TAT-Cx43_266–283_ strongly decreased their expression (white arrows indicate some examples; see also Supplementary Figure 5, in which the nuclear staining facilitates the distinction). Indeed, human nestin and Sox2 fluorescence levels were decreased by about 75% with TAT-Cx43_266–283_.

Reduced expressions of Sox2 and nestin suggest that TAT-Cx43_266–283_ decreases the stemness of the implanted GSCs in vivo. Therefore, we followed up the in vivo experiments for 30 days to analyze the tumorigenicity of GSCs. To identify human GSCs, in addition to PKH26, antibodies against the human antigen Stem121 were used^35^ and the expression of human nestin was analyzed as a marker of stemness in human xenografted cells. According to the reported GSC tumorigenicity,^37^ at 1 month post-implantation, we found GSCs that were able to engraft, proliferate, and migrate within the brain parenchyma in both TAT-treated (Fig. 4C, D) and control (Supplementary Figures 6, 7, 8) animals. However, TAT-Cx43_266–283_ reduced the number of PKH26-labeled and Stem121-positive cells 1 month after implantation (Fig. 4C and Supplementary Figure 7), suggesting reduced GSC survival and/or proliferation under these conditions. Indeed, the analysis of activated caspase-3 suggested an increased cell death in the tumor area after TAT-Cx43_266–283_ treatment, without effect in other brain areas, such as the subventricular zone (Supplementary Figure 9). Furthermore, those few remaining PKH26-labeled cells expressed low levels of human nestin (Fig. 4D and Supplementary Figure 8), suggesting the loss of stemness upon TAT-Cx43_266–283_ treatment. No significant differences were found when TAT treatment was compared with the control condition (Supplementary Figure 6).

### TAT-Cx43_266–283_ Prolongs the Survival of Glioma-Bearing Mice

**Fig. 5:**
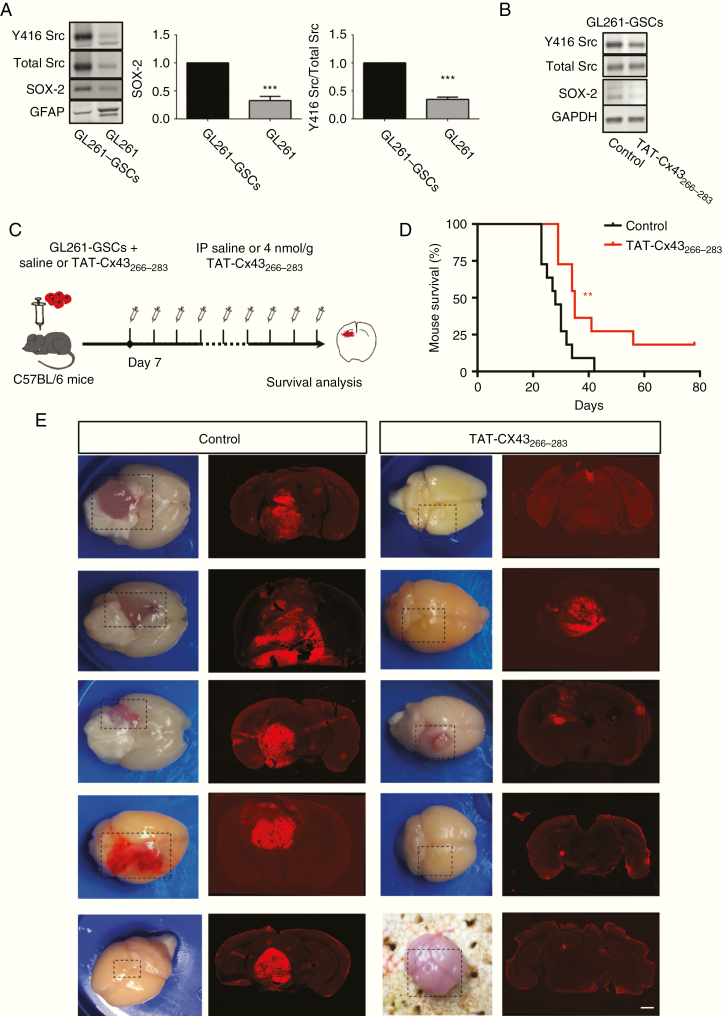
TAT-Cx43_266–283_ enhances the survival of immunocompetent mice bearing GL261-GSC-derived gliomas. (A) Western blot and quantification of Y416 Src, total Src, Sox2, and GFAP in GL261-GSCs and GL261 (Dulbecco’s modified Eagle’s medium + fetal calf serum for 7 days); means ± SEM; *n* = 8 (Student’s *t*-test: ***P < 0.001). (B) Western blot of Y416 Src, total Src, Sox2, and glyceraldehyde 3-phosphate dehydrogenase in GL261-GSCs control or treated with 50 µM TAT-Cx43_266–283_ for 24 h. (C) GL261-GSCs together with 100 µM TAT-Cx43_266–283_ or saline were intracranially injected in C57BL/6 mice. After 7 days, a twice per week i.p. injection of saline or 4 nmol/g TAT-Cx43_266–283_ was administered until neurological symptoms appeared. (D) Effect of TAT-Cx43_266–283_ on the survival of mice bearing orthotopic tumor syngrafts. Percentage of animals alive along the experiment depicted in Kaplan–Meier plot (*n* = 11 animals per condition from 3 independent experiments). Log-rank test **P < 0.01. (E) Representative images of the brains and tumor-bearing brain sections from control and treated animals at the end of the experiment. Bar: 1 mm.

To study the effect of TAT-Cx43_266–283_ on the survival of glioma-bearing mice, we combined the 2 in vivo models previously described. We isolated the highly tumorigenic subpopulation of GSCs from GL261 glioma cells (GL261-GSCs) because they generate more aggressive tumors with shorter mouse survival than their differentiated counterparts in an immunocompetent environment.^30,38^ Our results showed that GL261-GSCs exhibited higher Src activity and Sox2 levels than their differentiated counterpart GL261 (Fig. 5A), consistent with the role of Src in Sox2 expression and stemness.^11^ We confirmed that TAT-Cx43_266–283_ reduced both Src activity and Sox2 expression in GL261-GSCs in vitro (Fig. 5B), as shown in human GSCs.^18^ Then, GL261-GSCs were intracranially injected together with saline or TAT-Cx43_266–283_ into the brains of immunocompetent mice, and 1 week after tumor implantation mice were intraperitoneally injected with TAT-Cx43_266–283_ twice per week and followed for survival (Fig. 5C). A low amount of GL261-GSCs developed very aggressive tumors, characterized by their invasion, large size, angiogenesis, and very poor mice survival (Fig. 5D, E). Importantly, our results showed that TAT-Cx43_266–283_ significantly prolonged the survival of these animals (Fig. 5D), which exhibited tumors with reduced signs of aggressiveness (Fig. 5E).

## Discussion

We previously reported that the peptide based on Cx43, TAT-Cx43_266–283_, inhibits c-Src activity and exerts important effects in different types of glioma cells in vitro, including freshly removed surgical specimens of glioblastoma.^28^ In this study, we explored the possibility of using this peptide for therapy against malignant gliomas by studying its effect on healthy brain cells and by evaluating its antitumor effects in vivo.

The present study showed that the effect of  TAT-Cx43_266–283_ is cell selective. Thus, while GSC viability was strongly decreased, neuron and astrocyte viabilities were not greatly affected by TAT-Cx43_266–283_. Moreover, the morphology, expression of differentiation markers, and motility of these normal brain cells were unaffected by TAT-Cx43_266–283_. Conversely, TAT-Cx43_266–283_ reduced stemness, proliferation, survival, invasion, and migration in GSCs.^18,24,28^ The cell selectivity of TAT-Cx43_266–283_ might be due to a reduced rate of internalization, especially by neurons, compared with that of GSCs. The TAT peptide is composed mainly of positively charged amino acids, and its internalization relies on electrostatic interactions with the negative charges of the plasma membrane.^39^ One of the features of cancer cells, including GSCs, is a glycocalyx with a high content of negatively charged glycans, such as sialic acid, glucuronic acid, and glycosaminoglycans.^40,41^ This negatively charged glycocalyx could promote stronger electrostatic interactions with TAT-Cx43_266–283_ in GSCs compared with nontumor cells, contributing to the higher internalization. As expected for nontumor cells,^42^ c-Src activity is lower in astrocytes than in GSCs.^18^ Indeed, GSCs rely on the activity of this oncoprotein for survival, stemness, and invasion.^10^ Therefore, the cell selectivity of TAT-Cx43_266–283_ might depend both on its internalization and on the activity and function of c-Src. Thus, TAT-Cx43_266–283_ would specifically target cells harboring a negative glycocalyx with a high c-Src activity required for their survival.

The lack of TAT-Cx43_266–283_ toxicity found in neurons and astrocytes is in contrast to the results found with other Src inhibitors, such as dasatinib. Although TAT-Cx43_266–283_ and dasatinib promoted similar effects on GSC viability (as shown in this study) and stemness phenotype,^18^ TAT-Cx43_266–283_ showed lower toxicity than dasatinib in neurons and astrocytes. The mechanism of dasatinib inhibition of c-Src involves a hydrogen bond–mediated association with the ATP binding site, resulting in competitive restriction of ATP binding by c-Src.^36^ In addition to inhibiting c-Src, dasatinib also inhibits other members of the Src kinase family: BCR-ABL, c-KIT, PDGFR, and ephrin A2.^43^ However, TAT-Cx43_266–283_ inhibits c-Src by acting as a docking platform for c-Src together with its endogenous inhibitors CSK and PTEN.^24,44^ These data suggest that TAT-Cx43_266–283_, by using a specific and endogenous mechanism to inhibit c-Src activity, exerts lower toxicity in nontumor cells than other inhibitors that act through non-endogenous mechanisms with broader targets.

Intraperitoneally administered TAT-Cx43_266–283_ decreased the invasion of intracranial tumors generated by GL261 glioma cells in mice. This is the same in vivo glioma model used to show that Cx43, by increasing gap junctional communication, promotes invasion.^26^ Our results suggest that TAT-Cx43_266–283_ did not promote the deleterious increase in gap junctional communication between astrocytes and the GL261 Cx43-expressing glioma cells, because TAT-Cx43_266–283_ did not modify astrocytic gap junctional communication.^29^ However, our results suggest that TAT-Cx43_266–283_ impaired glioma invasion through inhibition of c-Src and FAK, as shown in vitro.^32^ These results indicate that the use of specific peptides would be a useful strategy for targeting specific connexin functions and a means to apply the vast connexin knowledge to the clinic.^27^ Our study shows that the effect of TAT-Cx43_266–283_ on cell invasion is not restricted to GSCs but is also significant at the bulk tumor level.

Our study reveals that intracranial injection of TAT-Cx43_266–283_ with GSCs reduced the expression of nestin and Sox2, crucial markers of stemness,^45,46^ in GSCs at 7 days post-implantation. Consistent with the role of Sox2 as a transcription factor required for GSC tumorigenicity,^47^ we found that TAT-Cx43_266–283_ strongly decreased GSC tumorigenicity, as judged by the lower number of human glioma cells found 1 month post-implantation. In fact, TAT-Cx43_266–283_ importantly reduced not only the number of human glioma cells, but also their expression of nestin, suggesting that the remaining glioma cells did not exhibit stem cell properties known to drive recurrence. Furthermore, the survival of immunocompetent mice bearing gliomas derived from murine glioma stem cells was enhanced when these animals were treated with TAT-Cx43_266–283_.

Altogether, these data confirm the relevance of TAT-Cx43_266–283_ for the design of new therapies against gliomas. Indeed, after a thorough investigation that included in vitro and in vivo models, such as intracranial implantation of murine glioma cells and GSCs into immunocompetent mice and of human GSCs into immunosuppressed mice, we can conclude that TAT-Cx43_266–283_ prolongs the survival of glioma-bearing mice because it reduces the growth, invasion, and progression of malignant gliomas with remarkably fewer toxic effects for normal brain cells than other c-Src inhibitors currently being tested in glioma clinical trials. It should be highlighted that TAT peptides have been proven to be safe and effective for stroke in preclinical models^48^ and in patients after intravenous administration^49^ and are currently being evaluated in a phase III clinical trial (https://clinicaltrials.gov/ct2/show/NCT02930018). These studies confirm the ability of peptides fused to TAT to cross the human blood–brain barrier and the suitability of the systemic administration of these compounds for CNS therapy. Therefore, intravenous administration of TAT-Cx43_266–283_ might be efficient for malignant gliomas. Because most patients with malignant glioma show recurrence after surgery within or in close proximity to the original site,^50^ another attractive proposal would be to explore the local administration of TAT-Cx43_266–283_ within the postoperative cavity through a sustained-release drug delivery system, in combination with standard therapies. According to our results, TAT-Cx43_266–283_ might reduce tumor cell invasion and could target those GSCs that escape the surgery, reversing their stemness and consequently reducing their tumorigenic activity, preventing the regrowth of the tumor. Further research is required to explore this interesting possibility and its future application in the clinic.

We are aware that most preclinical positive results in the field of malignant gliomas failed when applied in the clinic. However, the effects of TAT-Cx43_266–283_ in GSCs and at the bulk tumor level, the positive results in freshly removed surgical specimens of glioblastoma patients^28^ as well as in immunocompetent in vivo models, and the lack of toxicity suggest that the combination of this compound with existing therapies could improve the survival of these patients.

## Supplementary Material

noz243_suppl_Supplementary_fig_S1

noz243_suppl_Supplementary_fig_S2

noz243_suppl_Supplementary_fig_S3

noz243_suppl_Supplementary_fig_S4

noz243_suppl_Supplementary_fig_S5

noz243_suppl_Supplementary_fig_S6

noz243_suppl_Supplementary_fig_S7

noz243_suppl_Supplementary_fig_S8

noz243_suppl_Supplementary_fig_S9

noz243_suppl_Suplementary_movie_S1

noz243_suppl_Suplementary_movie_S2

noz243_suppl_Suplementary_movie_S3

noz243_suppl_Suplementary_movie_S4

noz243_suppl_Suplementary_Information
