# Erratum to ‘Connexin43 peptide, TAT-Cx43_266–283_, selectively targets glioma cells, impairs malignant growth, and enhances survival in mouse models in vivo’

**DOI:** 10.1093/neuonc/noaa087

**Published:** 2020-06-09

**Authors:** Myriam Jaraíz-Rodríguez, Rocío Talaverón, Laura García-Vicente, Sara G Pelaz, Marta Domínguez-Prieto, Andrea Álvarez-Vázquez, Raquel Flores-Hernández, Wun Chey Sin, John Bechberger, José M Medina, Christian C Naus, Arantxa Tabernero

In the original version of this article, the placement of asterisk was inadvertently missed in figure 2E and 5D. This has been corrected and the correct figures appear online. The publisher regrets the error.

