# Supplementary figures and images for: Connexin43 peptide, TAT-Cx43266–283, selectively targets glioma cells, impairs malignant growth, and enhances survival in mouse models in vivo
Source: Neuro Oncol. 2019 Dec 28;22(4):493–504. doi: 10.1093/neuonc/noz243 (PMC7158688; doi:10.1093/neuonc/noz243)

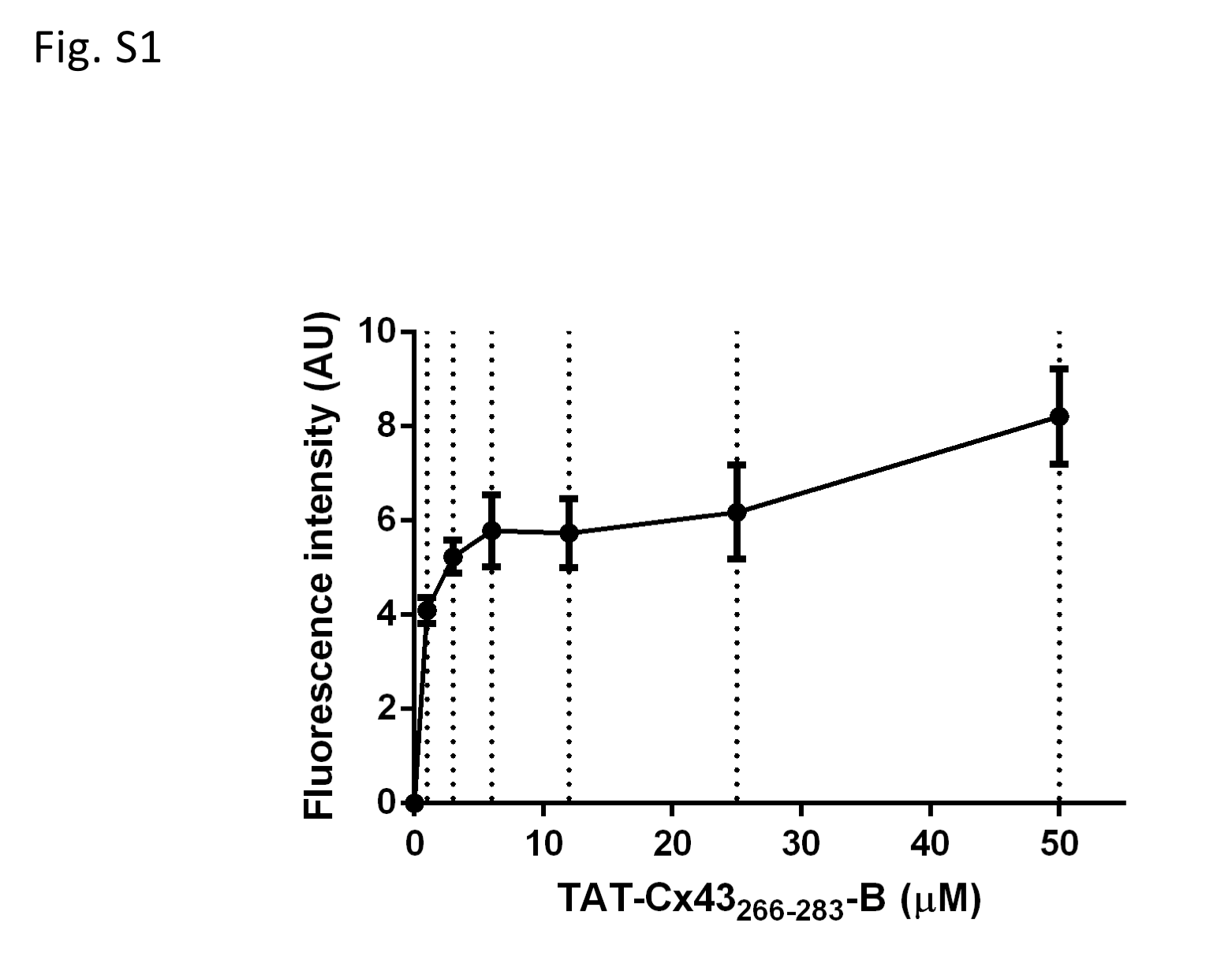

Supplement: noz243_suppl_Supplementary_fig_S1 [file noz243_suppl_supplementary_fig_s1.png]

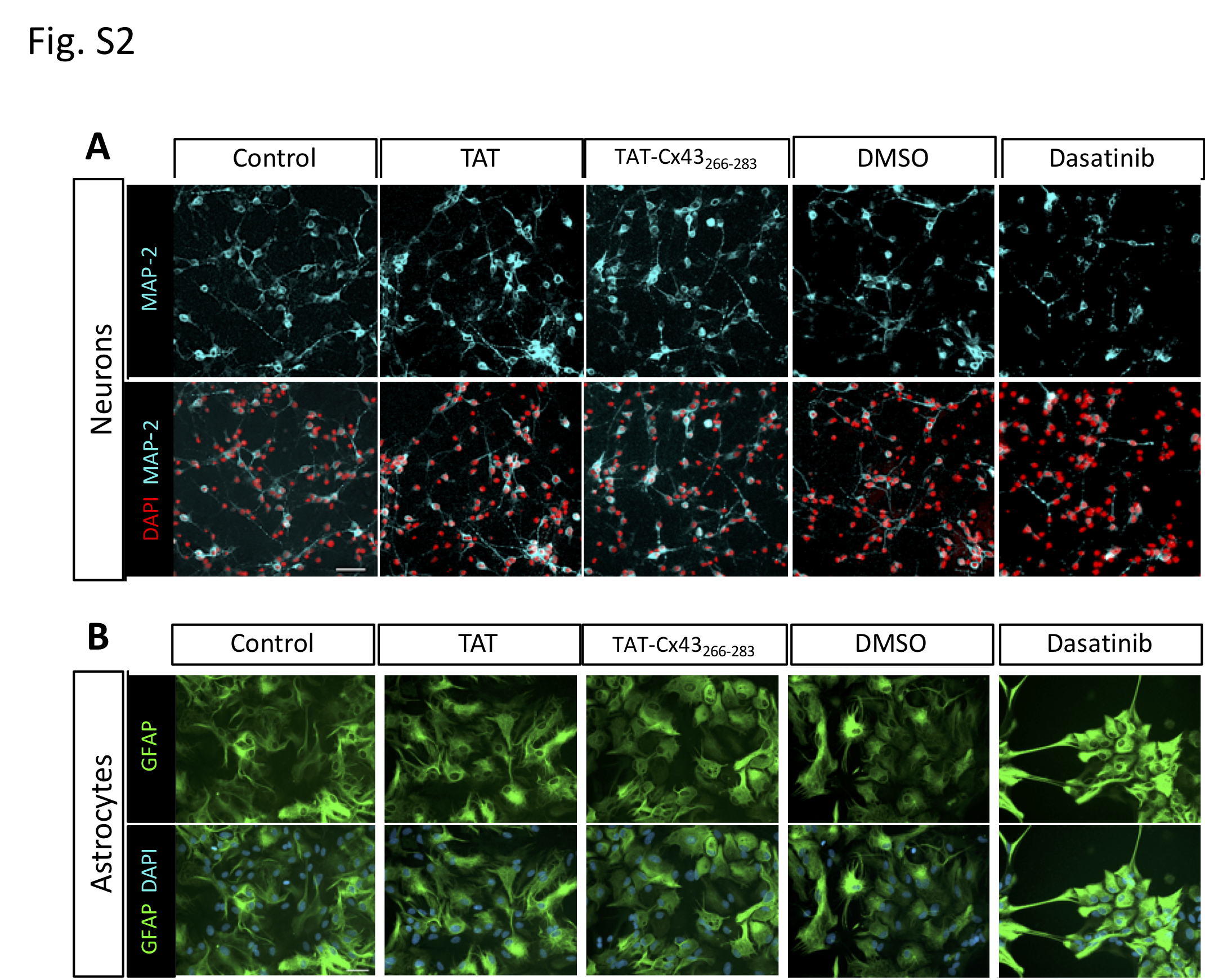

Supplement: noz243_suppl_Supplementary_fig_S2 [file noz243_suppl_supplementary_fig_s2.png]

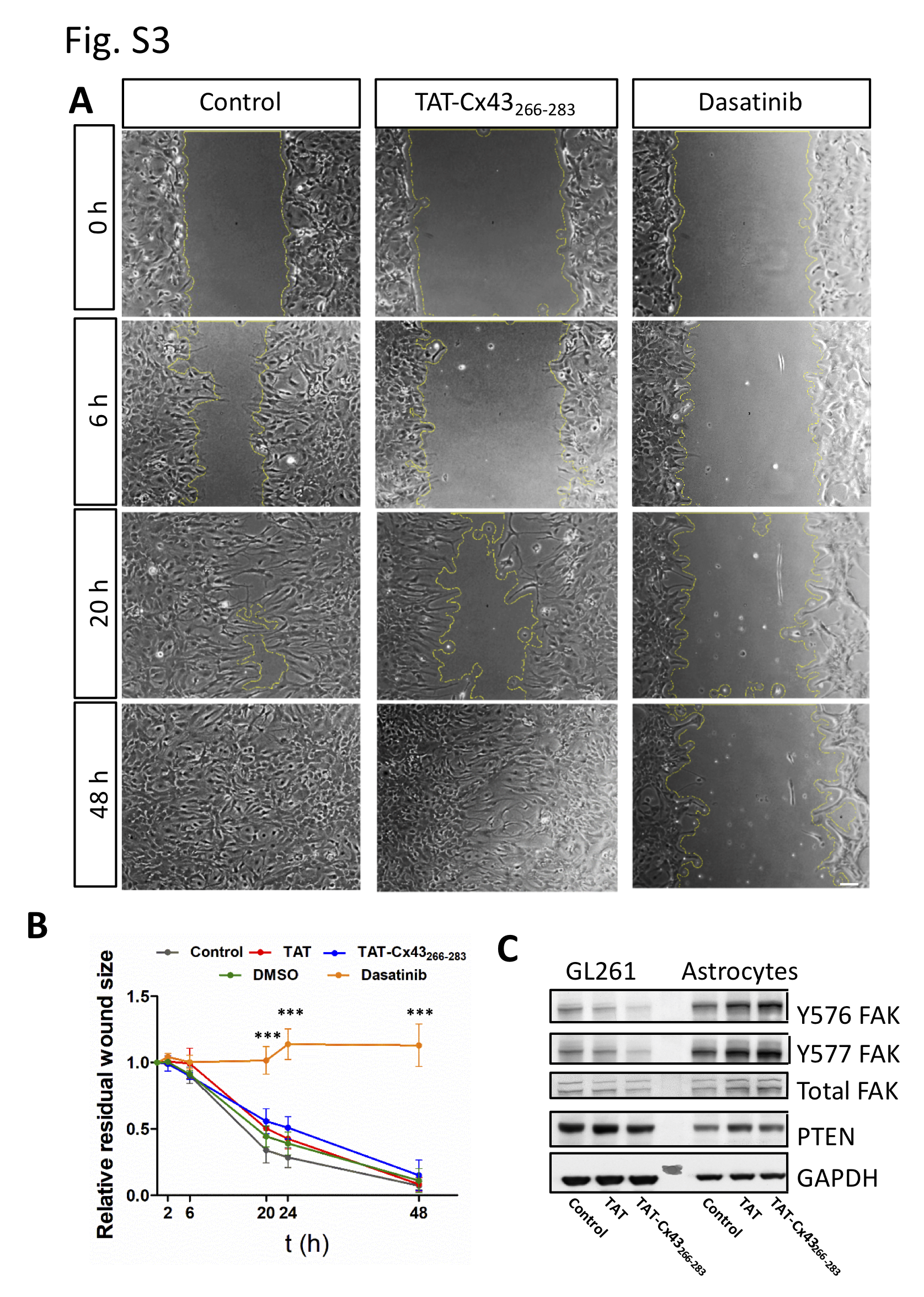

Supplement: noz243_suppl_Supplementary_fig_S3 [file noz243_suppl_supplementary_fig_s3.png]

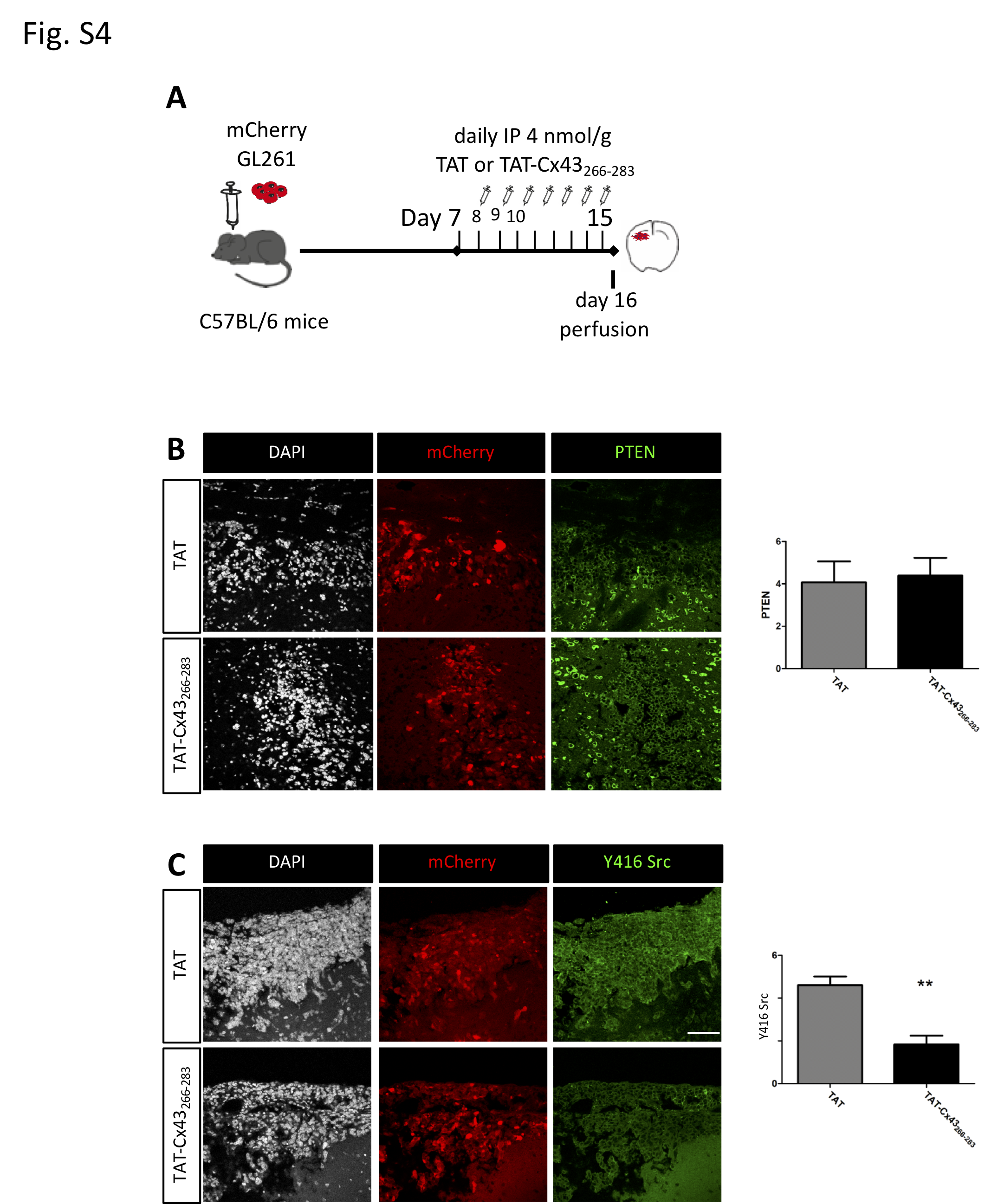

Supplement: noz243_suppl_Supplementary_fig_S4 [file noz243_suppl_supplementary_fig_s4.png]

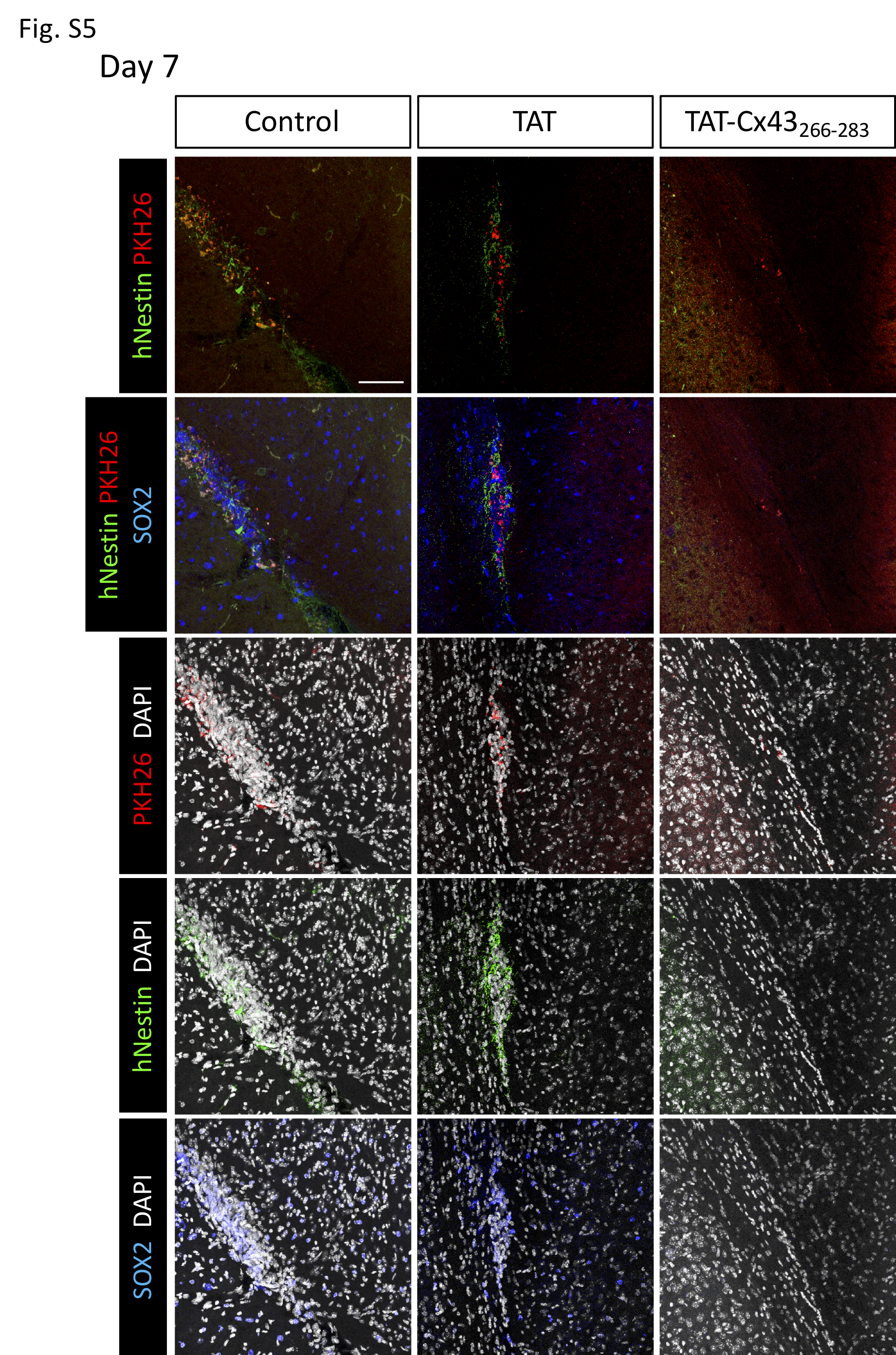

Supplement: noz243_suppl_Supplementary_fig_S5 [file noz243_suppl_supplementary_fig_s5.png]

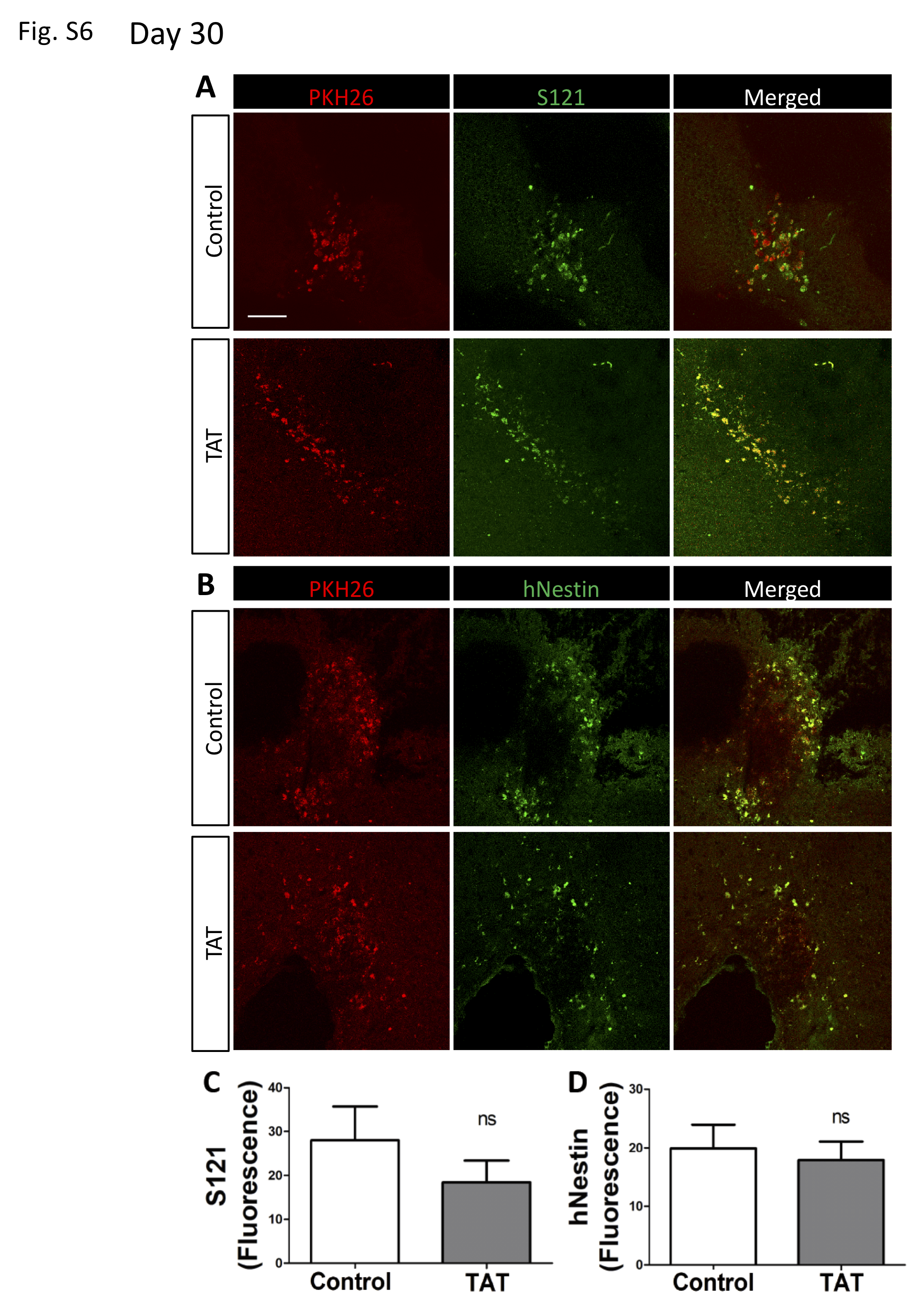

Supplement: noz243_suppl_Supplementary_fig_S6 [file noz243_suppl_supplementary_fig_s6.png]

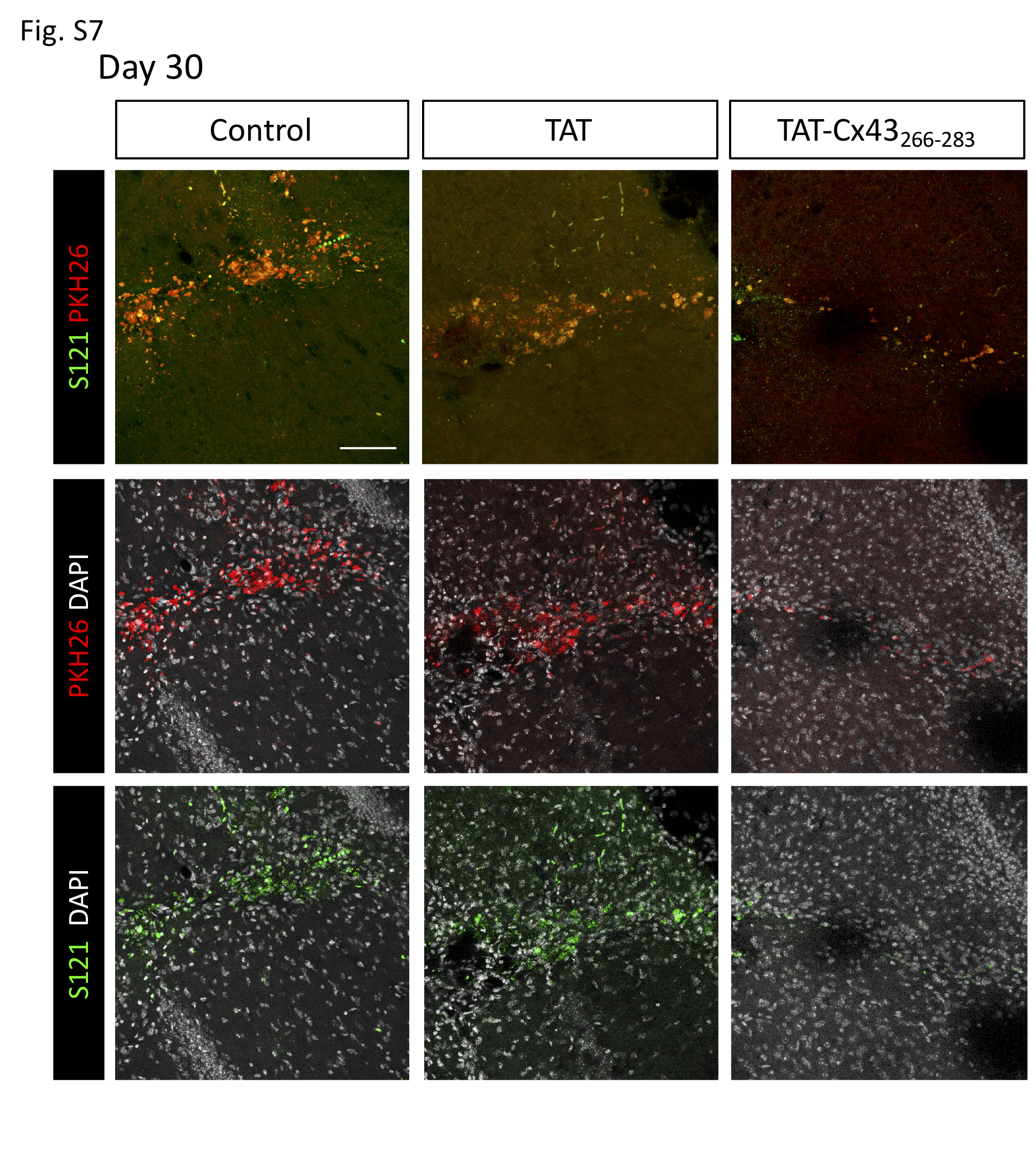

Supplement: noz243_suppl_Supplementary_fig_S7 [file noz243_suppl_supplementary_fig_s7.png]

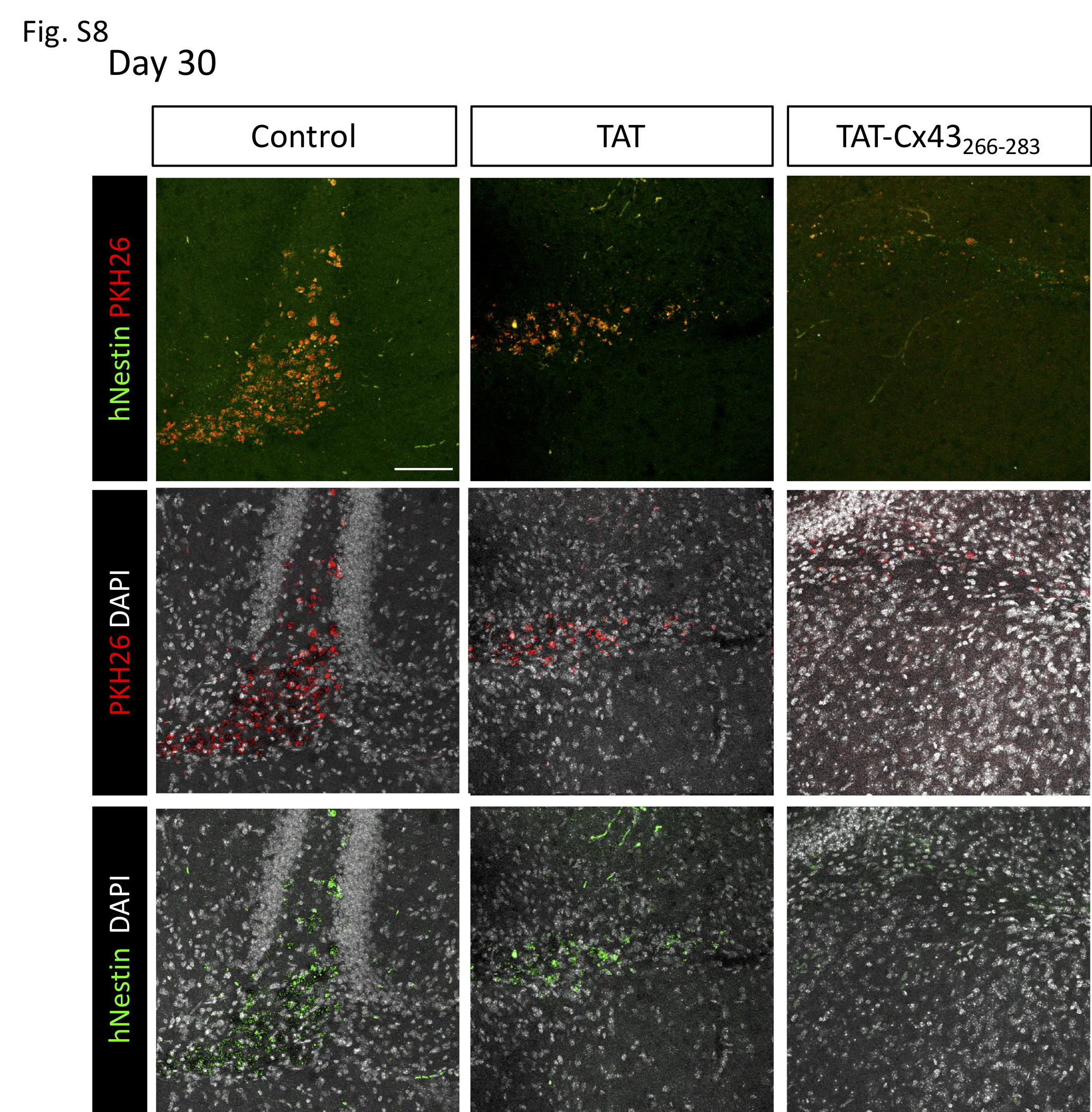

Supplement: noz243_suppl_Supplementary_fig_S8 [file noz243_suppl_supplementary_fig_s8.png]

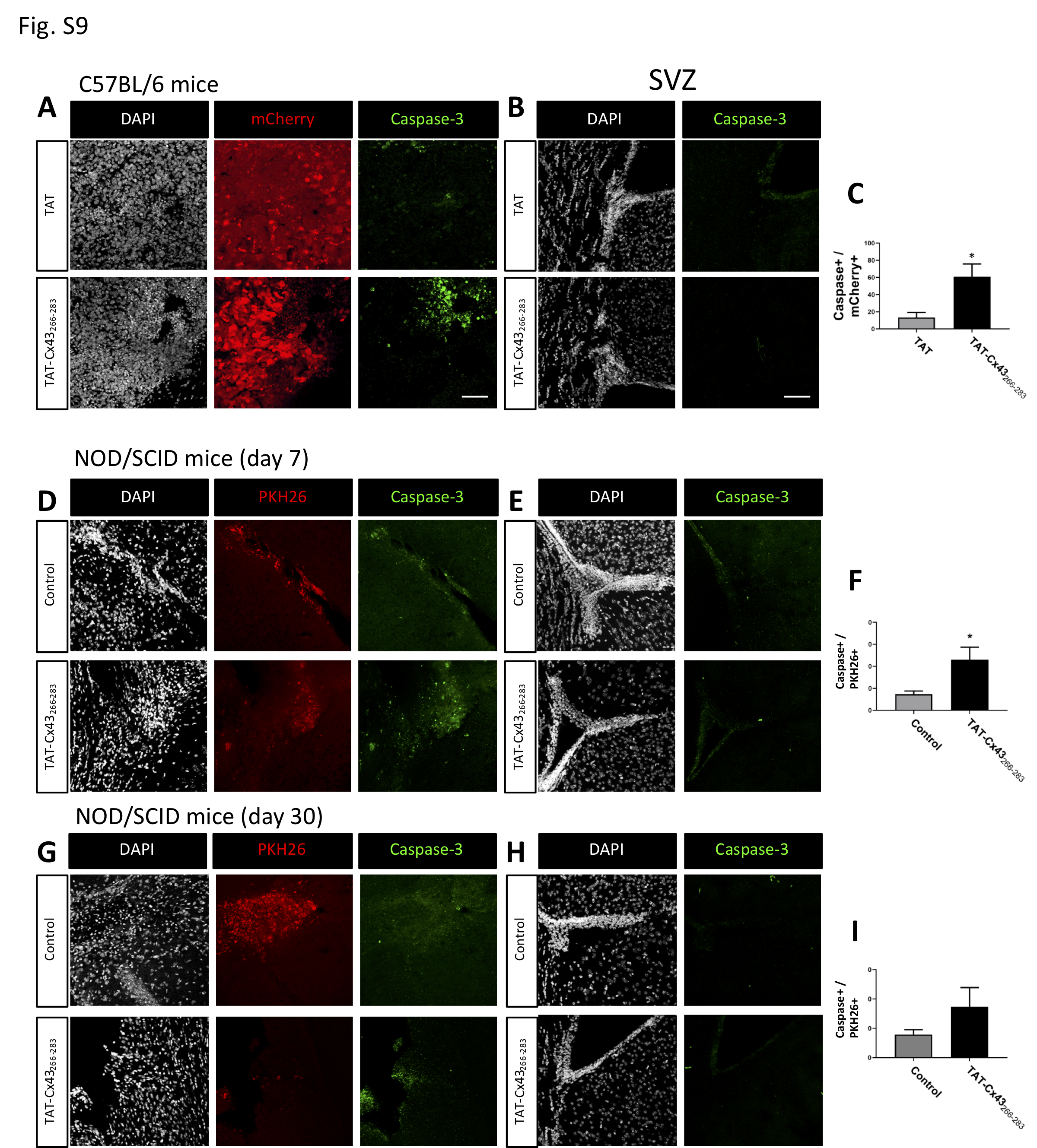

Supplement: noz243_suppl_Supplementary_fig_S9 [file noz243_suppl_supplementary_fig_s9.png]
