# Supplementary material for: Connexin43 peptide, TAT-Cx43266–283, selectively targets glioma cells, impairs malignant growth, and enhances survival in mouse models in vivo
Source: Neuro Oncol. 2019 Dec 28;22(4):493–504. doi: 10.1093/neuonc/noz243 (PMC7158688; doi:10.1093/neuonc/noz243)
Supplement: noz243_suppl_Suplementary_Information [file noz243_suppl_suplementary_information.docx]

**Supplementary Information**

**Supplementary Methods**

**Cell culture**

Primary astrocytes were prepared from the forebrains of 1- to 2-day-old Wistar rats and primary neurons from the forebrains of fetuses at 17.5 days of gestation.

Culture medium for neurons, astrocytes and GL261 glioma cells was Dulbecco’s modified Eagle’s medium (DMEM D5523; Sigma-Aldrich, Madrid, Spain) supplemented with 10% fetal calf serum (FCS; Gibco, Life Technologies, Madrid, Spain).

Culture medium for human G166 GSCs was RHB A medium (Takara Bio Inc., Condalab, Madrid, Spain) supplemented with 2% B27, 1% N2 (Life Technologies), 20 ng/mL EGF, and 20 ng/mL b-FGF (PeproTech, London, UK). Culture plates were coated with 10 µg/mL laminin (Natural Mouse Laminin, Life Technologies) up to 2 h before use. G166 GSCs were grown to confluence, dissociated using Accutase (Sigma-Aldrich), and then split as convenient. G166 GSCs were routinely expanded for no more than 15 passages.

GL261-GSCs were obtained from GL261 cells by decreasing one half of FCS concentration every 3 days^30^. They were cultured as spheres in stem cell medium containing Dulbecco’s modified Eagle’s Medium/Nutrient Mixture F-12 Ham (Sigma-Aldrich) supplemented with 1% Minimum Essential Medium-Non-Essential Amino Acids (MEM-NEAA; Gibco), 3.9 mM glucose, 1 mM L-glutamine, 0.07% β-mercaptoethanol (Sigma-Aldrich), 121.8 μg/mL bovine serum albumin (BSA; Roche Diagnostics, Sant Cugat del Vallès, Spain), 1% B27, 0.5% N2, 10ng/mL EGF and 20 ng/mL b-FGF. GL261-GSC spheres were dissociated using Accutase and subcultured as required every 10 to 14 days. For GL261-GSC differentiation, they were cultured in DMEM containing 10% FCS for 7 days.

Cell cultures were maintained at 37 °C in an atmosphere of 95% air/5% CO_2_ and with 90%–95% humidity.

**Organotypic brain slice cultures**

Culture medium was DMEM (Sigma-Aldrich) supplemented with 10% horse serum and glucose (33.3 mM final concentration). For GSC-organotypic brain slice co-cultures, culture medium was RHB-A supplemented with 2% B27, 1% N2, 20 ng/mL EGF, and 20 ng/mL b-FGF.

**Intracranial implantation of glioma cells**

Equal numbers of adult male and female mice were used for these experiments.

Mice were anesthetized by isoflurane inhalation, placed on a stereotaxic frame, and window-trephined in the parietal bone. Cell suspensions were kept on ice during the surgery.

Injection coordinates were: 5 mm caudal to Bregma, 4 mm lateral, and at a 2 mm depth. To minimize the inflammatory response from damaged brain tissue due to the needle injection, tumoral cells were slowly injected into the brain and the needle was held in place for an additional 2 min before removal.

For perfusion, mice were anesthetized with pentobarbital 120 mg/kg, 0.2 mL.

Transcardially perfusions were performed with 15 mL of physiological saline followed by 25 mL of 4% paraformaldehyde in 0.1 M phosphate buffer, pH 7.4. Brains were removed and cryoprotected by immersion in a solution of 30% sucrose in PBS until they sank. Then, 20–40-µm-thick coronal sections were obtained with a cryostat to be processed for immunostaining. Z-stack confocal images were taken at 40× and their maximum Z projection is shown in the figures.

**Treatments**

YGRKKRRQRRR was used as the TAT sequence, which is responsible for the cell penetration capability of the peptides^31^. The TAT-Cx43_266-283_ sequence was TAT-AYFNGCSSPTAPLSPMSP. The sequence for biotinylated TAT-Cx43-_266-283_ (TAT-Cx43-_266-283_-B) was TAT-AYFNGCSSPTAPLSPMSP-Lys(biotin).

The c-Src inhibitor dasatinib was dissolved in DMSO (stock solution, 1 mM) and used at 1 µM in culture medium at 37 °C for the indicated times. The control consisted of 0.1% (v/v) DMSO.

For in vivo studies in C57BL/6 mice, at day 8, 4 nmol/g/day of saline, TAT or TAT-Cx43_266-283_ was intraperitoneally injected daily for the next 7 days. For survival experiments, intracranial injection of 1 µL saline containing 5,000 GL261-GSCs in the absence or presence of 100 µM TAT-Cx43_266-283_ was performed. At day 8, 4 nmol/g/day of saline, or TAT-Cx43_266-283_ was intraperitoneally injected twice per week until neurological symptoms became evident.

For in vivo studies in NOD/SCID mice, one single intracranial injection of 1 µL saline containing 100 µM TAT or 100 µM TAT-Cx43_266-283_ and 5,000 GSCs was performed.

**Immunofluorescence**

Cells or organotypic slices were fixed in 4% paraformaldehyde for 20 min. They were then rinsed in PBS and incubated for 1 h at 25 °C (cells) or for 12 h at 4 °C (organotypic slices) in blocking solution (PBS containing 10% FCS, 0.1 M lysine, and 0.02% azide) with 0.1% Triton X-100. The samples were incubated overnight at 4 °C with one of the following primary antibodies: mouse monoclonal antibody against GFAP (clone G-A-5; 1:500; Sigma-Aldrich, Ref. G3893), rabbit polyclonal antibody against GFAP (1:500; Sigma-Aldrich, Ref. G9269), mouse monoclonal antibody against MAP-2 (clone AP-20; 1:500; Sigma-Aldrich, Ref. M1406), mouse monoclonal antibody against human nestin (clone 2C1.3A11; 1:200; Abcam, Cambridge, UK, Ref. ab18102). After repeated washes, they were incubated with the corresponding secondary antibody for 75 min: anti-rabbit or anti-mouse IgG conjugated with Alexa Fluor 488, 594, or 647 (1:1,000; Life Technologies). Antibodies were prepared in blocking solution with 0.1% Triton X 100. Finally, nuclear DNA was stained with 1 µg/mL 4’,6-diamidino-2-phenylindole (DAPI) for 1 min or 1:1,000 TO-PRO-3 (Life Technologies) for 10 min. To detect TAT-Cx43_266-283_-B_,_ Cy2-conjugated streptavidin (1:500 for cells and 1:1,000 for organotypic slices; Jackson ImmunoResearch, Baltimore, MD, USA) was applied for 75 min. Cells were mounted using a SlowFade Light antifade kit (Life Technologies) and analyzed on an inverted fluorescence microscope Leica connected to a digital video camera (Leica DC 100; Leica Microsystems, Wetzlar, Germany), an inverted Zeiss Axio Observer Z1 microscope for live-cell imaging (Carl Zeiss Microscopy, LLC, Thornwood, NY, USA) coupled to an AxioCam MRm camera, or a Leica TCS SP2 confocal microscope.

For in vivo studies, sections were washed in PBS and incubated with 1% sodium borohydride or fresh citrate buffer (pH=6) for 10 min for antigen retrieval. Sections were rinsed again in PBS and incubated for 1 or 2h in blocking solution. Then, sections were incubated overnight at room temperature with one of the following primary antibodies prepared in blocking solution: mouse monoclonal antibody against human nestin (1:500; Abcam, Ref. ab18102), rabbit polyclonal anti-SOX-2 (1:200; Abcam, Ref. ab97959), mouse monoclonal anti-stem121 (1:100; Takara, Ref. Y40410), rabbit polyclonal antibody anti-caspase 3 (1:200; Cell Signaling, Ref.9661S), rabbit polyclonal antibody anti-PTEN (1:100; Abcam, Ref.ab137337) and rabbit polyclonal antibody anti Src (Tyr 416) (1:50; Cell Signaling, Ref. 2101). After repeated washes, sections were incubated for 2 h with the corresponding secondary antibody prepared in PBS with 0.1% Triton X-100: anti-rabbit or anti-mouse IgG conjugated with Alexa Fluor 488 or 647 (1:500; Life Technologies). Finally, nuclear DNA was stained with 1 µg/mL DAPI for 1 min or 1:1,000. Sections were washed, mounted with SlowFade, and analyzed by confocal microscopy (Leica TCS SP2 and Leica SP5). Different focal planes (2 µm along the Z axis) were scanned with a pinhole aperture of 1 Airy unit and their maximum Z projection is shown in the figures.

**Fluorescence quantification**

Images were analyzed using Fiji open source software, available at <http://fiji.sc>. The fluorescence intensity was quantified as the mean gray value per region of interest (region occupied by PKH26-labeled cells) in the maximum Z projection confocal images. Non-specific background fluorescence was subtracted in all the images analyzed. For peptide internalization experiments, fluorescence intensity was quantified as integrated density (IntDen value) per number of cells and expressed in arbitrary units (AU).

The quantification of the invasiveness of intracranial tumors was performed with FracLac (a Fiji plugin to analyze difficult-to-describe morphological features).

**Time-lapse microscopy**

Primary rat neurons were plated at 100,000 cells/cm^2^ in 24-well plates. After 6 h, 50 µM TAT or TAT-Cx43_266-283_ was added and the plates were allowed to equilibrate for 1 h in the microscope incubator before imaging.

Phase-contrast photomicrographs of random fields were taken every 10 min by time-lapse live-cell imaging for 48 h with an inverted microscope.

The microscope was an inverted Zeiss Axio Observer Z1 for Live-Cell Imaging. The system includes an automated XY stage controller and a humidified incubator set at 37 °C and 5% CO_2_. Image stacks were processed using Zen imaging software (Carl Zeiss Microscopy).

**Wound-healing assays**

A scratch was made in confluent astrocyte monolayers using a yellow pipette tip. Then, cells were treated with 50 µM TAT, 50 µM TAT-Cx43_266-283_, or 1 µM dasatinib (or 1 µL/mL of DMSO vehicle). Phase-contrast photomicrographs were taken at 0, 2, 6, 20, 24, and 48 h after the scratch was made on a Leica inverted fluorescence microscope connected to a digital video camera (Leica DC 100; Leica Microsystems). Images were analyzed using the MRI wound-healing tool (Fiji).

**Western blotting**

Western blotting was performed as previously described (Herrero-Gonzalez et al., 2010). Briefly, equivalent amounts of proteins were separated on NuPAGE Novex Bis-Tris (4-12%) midigels (Life Technologies). The proteins were transblotted using an iBlot dry blotting system (Life Technologies). After blocking, the membranes were incubated overnight at 4 ºC with the primary antibodies against GFAP (1:1000; Sigma, Ref. G9269), SOX-2 (1:500; Abcam, Ref. ab97959), Y416 Src (1:200; Cell Signaling, Danvers, MA, USA; Ref. 2101), total Src (1:500; Cell Signaling; Ref. 2108), PTEN (1:500; Cell Signaling; Ref. 9556S), Y576 FAK (1:500; Life Technologies; Ref. 44652G), Y577 FAK (1:500; Life Technologies; Ref. 44-614G) and total FAK (1:500; Life Technologies; Ref. AHO0502). Glyceraldehyde phosphate dehydrogenase (GAPDH, 1:5000; Ambion, Thermo Fisher Scientific; Ref. AM4300) or alpha-actinin (1:1000; Chemicon International, Merck Millipore; Ref. MAB1682) were used as a loading control. After extensive washing, the membranes were incubated with peroxidase-conjugated anti-rabbit IgG or anti-mouse IgG antibodies (Santa Cruz Biotechnology, Inc., Dallas, TX, USA; Refs. sc-2030 and sc-2005) in TTBS and developed with a chemiluminescent substrate (Western blotting Luminol Reagent; Santa Cruz Biotechnology) in a MicroChemi imaging system (Bioimaging Stystems) or X-ray ﬁlms obtained from Fujiﬁlm (Madrid, Spain). Densitometry analysis of the bands was performed using Fiji software.

**Matrigel invasion assay**

Transwell inserts (Merck Millipore, Madrid, Spain) containing polyethylene terephthalate filters with 8-μm pores were coated with 100 µL of 1 mg/mL Matrigel matrix (Corning, Amsterdam, the Netherlands) according to the manufacturer’s recommendations. Cells were cultured in 200 µL of serum-free medium in the upper chamber while 500 µL of medium supplemented with 10% FCS was added to the lower well. The invading cells were fixed with 4% paraformaldehyde for 10 min, washed with PBS, and stained with Giemsa for 10 min. Images were taken using a Leica microscope connected to a digital camera (Leica DFC500).

**Quantification of the invasiveness of intracranial tumors**

Twenty-micron-thick cryosections were blocked for 1 h at room temperature in 2% bovine serum albumin and 0.3% Triton X-100. Sections were then incubated overnight at 4 °C in 1% bovine serum albumin and 0.1% Triton X-100, mounted in Prolong antifade mounting media (Invitrogen) with DAPI, and visualized by confocal microscopy with a Leica TCS SP5 II Basic VIS system (Leica Microsystems, Concord, ON, Canada).

### **Figure legends for supplementary figures and videos**

**Figure 1S. Dose-response of TAT-Cx43_266-283_ internalization in GSCs.** GSCs were incubated with increasing concentrations of TAT-Cx43_266-283_ fused to biotin (TAT-Cx43_266-283_-B) as described in Figure 1. After 5 min, cells were fixed and incubated with fluorescent streptavidin and the fluorescence intensity was quantified. The results are expressed as the mean ± s.e.m. of three independent experiments.

**Figure 2S. Merged images, including DAPI, of MAP-2 and GFAP in neurons and astrocytes.** Cultured neurons, or astrocytes were treated with 50 µM TAT, 50 µM TAT-Cx43_264-283_, 1 µM of the c-Src inhibitor dasatinib, or 0.1% (v/v) DMSO (vehicle for dasatinib) as described in figure 2. After 72 h, cells were analyzed by microscopy. (**A**) Immunofluorescence for MAP-2 (turquoise) and merged images including DAPI (red) of the same fields. Bar: 50 µm. (**B**) Immunofluorescence images for GFAP (green) and merged images including nuclear staining with DAPI (turquoise) of the same fields. Bar: 50 µm.

**Figure 3S. Effect of TAT-Cx43_266-283_ on astrocyte migration and FAK activity.** Wound-healing assay in monolayers of astrocytes. After the wound was made, phase-contrast photomicrographs were taken (t=0). Then, cells were treated with 50 µM TAT, 50 µM TAT-Cx43_264-283_, 1 µM of the c-Src inhibitor dasatinib, or 0.1% (v/v) DMSO (vehicle for dasatinib) and phase-contrast photomicrographs were taken at the indicated times. (**A**) Phase-contrast images showing the evolution of the healing. Bar: 100 µm. (**B**) Quantification of the residual wound size relative to the size found at time 0 expressed as the mean ± s.e.m. of three experiments (ANOVA: ***p<0.001). (**C**) Astrocytes and Gl261 cells were incubated with 50 µM TAT or 50 µM TAT-Cx43_266-283_. After 24 h, PTEN, total FAK and Y576 and Y577 FAK levels were analyzed by western blotting. GAPDH was used as control.

**Figure 4S.** **Effect of TAT-Cx43_266-283_ on the levels of Y416 Src and PTEN in tumors generated by GL261 glioma cells in vivo.** (**A**) GL261 cells were intracranially implanted in syngeneic mice (C57BL/6) and allowed to grow for 7 days. Then, a daily IP injection of 4 nmol/g of TAT or TAT-Cx43_266-283_ was administered for the next 7 days as described in Figure 3. (**B**) Representative images of brain sections showing Dapi (gray), mCherry (red), and PTEN (green). Quantification of PTEN fluorescence intensity within the tumor area. (**C**) Representative images of brain sections showing DAPI (gray) mCherry (red) and Y416 Src (green). Quantification of Y416 Src fluorescence intensity in 16 positive mCherry cells per field. Bar: 75 µm. The results are expressed as mean ± s.e.m. Three sections per animal and three animals per condition were analyzed to perform a statistical comparison (Student’s t test: **p<0.01).

**Figure 5S. Merged images, including DAPI, of** **SOX-2 and human nestin in human GSCs intracranially implanted into mice for 7 days.** Representative images from the experiments described in Figure 4B including nuclear staining with DAPI. Briefly, 5,000 human GSCs labeled with PKH26 were intracranially injected in the absence (control) or presence of 100 µM TAT or TAT-Cx43_266-283_ into NOD/SCID mice. After 7 days, brains were processed. Images showing PKH26 (red), human nestin (hNestin; green), SOX-2 (blue), and merged images of the same field including nuclear staining with DAPI (white). Bar: 75 µm.

**Figure**  **6S. Effect of TAT on the expression of S121 and human nestin in human GSCs intracranially implanted into mice for 30 days.** Briefly, 5,000 human GSCs labeled with PKH26 were intracranially injected in the absence (control) or presence of 100 µM TAT into NOD/SCID mice for 30 days. (**A** and **B**) Representative images showing PKH26 (red), Stem121 (S121, A), and human nestin (hNestin, B). Bar: 75 µm. (**C** and **D**) Quantification of S121 (C) and hNestin (D) fluorescence intensity. The results are the mean ± s.e.m. Between 2 and 5 sections per animal and 4 control and 5 TAT animals from three independent experiments were analyzed (Student’s t test: non-significant p-value).

**Figure 7S. Merged images, including DAPI, of** **S121 in human GSCs intracranially implanted into mice for 30 days.** Representative images from the experiments described in Figure 4C including nuclear staining with DAPI. Briefly, 5,000 human GSCs labeled with PKH26 were intracranially injected in the absence (control) or presence of 100 µM TAT or TAT-Cx43_266-283_ into NOD/SCID mice for 30 days. Representative images showing PKH26 (red), stem121 (S121; green), and merged images of the same field including nuclear staining with DAPI (white). Bar: 75 µm.

**Figure 8S. Merged images, including DAPI, of** **human nestin in human GSCs intracranially implanted into mice for 30 days.** Representative images from the experiments described in Figure 4D including nuclear staining with DAPI. Briefly, 5,000 human GSCs labeled with PKH26 were intracranially injected in the absence (control) or presence of 100 µM TAT or TAT-Cx43_266-283_ into NOD/SCID mice for 30 days. Representative images showing PKH26 (red), human nestin (hNestin; green), and merged images of the same field including nuclear staining with DAPI (white). Bar: 75 µm.

**Figure 9S. Effect of TAT-Cx43_266-283_ on glioma cell death**. Immunofluorescence for activated caspase-3 in brain sections obtained as described in Figure 3 (**A-C**), Figure 4B (**D-F**) and Figure 4C (**G-I**). (**A**, **D**, and **G**) Representative images showing the increase in activated caspase-3 staining (green) in glioma cells (red; PKH26 or mCherry) after TAT-Cx43_266-283_ treatment. (**B**, **E**, and **H**) Representative images showing the lack of effect of TAT-Cx43_266-283_ in activated caspase-3 staining (green) in the subventricular zone (SVZ). (**C**, **F**, and **I**) Number of caspase-3 positive glioma cells per section. Bar: 75 µm. The results are the mean ± s.e.m. 2-5 sections per animal and 3 animals per condition were analyzed (Student’s t test: *p<0.05).

**Video S1:** **TAT-Cx43_266-283_ internalization in GSC–organotypic brain slice co-culture.** The complete Z-stack from the image shown in Figure 1E.

**Video S2:** **Time-lapse movie showing the development of rat primary neurons.** Primary rat neurons were plated at 100,000 cells/cm^2^ and cultured (control). Phase-contrast time-lapse movies were taken for 48 h as described in the Methods. Frame size: 720 × 480 pixels. Frame rate: 48 frames per second.

**Video S3: Time-lapse movie showing the effect of TAT on the development of rat primary neurons.** Primary rat neurons were plated at 100,000 cells/cm^2^ and cultured in the presence of 50 µM TAT. Phase-contrast time-lapse movies were taken for 48 h as described in the Methods. Frame size: 720 × 480 pixels. Frame rate: 48 frames per second.

**Video S4: Time-lapse movie showing the effect of TAT-Cx43_266-283_ on the development of rat primary neurons.** Primary rat neurons were plated at 100,000 cells/cm^2^ and cultured in the presence of 50 µM TAT-Cx43_266-283_. Phase-contrast time-lapse movies were taken for 48 h as described in the Methods. Frame size: 720 × 480 pixels. Frame rate: 48 frames per second.
